# A Wi-Fi-Based Wireless Indoor Position Sensing System with Multipath Interference Mitigation

**DOI:** 10.3390/s19183983

**Published:** 2019-09-14

**Authors:** Tuo Xie, Hanjun Jiang, Xijin Zhao, Chun Zhang

**Affiliations:** Institute of Microelectronics, Tsinghua University, Beijing 100084, China; xie-t12@mails.tsinghua.edu.cn (T.X.); zhaoxj@tsinghua.edu.cn (X.Z.); zhangchun@tsinghua.edu.cn (C.Z.)

**Keywords:** indoor position sensing, RSSI, TDoA, multipath interference, Wi-Fi

## Abstract

Wi-Fi-based indoor position sensing solutions have the advantages of easy integration in mobile phones and low cost by using existing Wi-Fi access points. The mainstream methods are commonly based on the received signal strength indicator (RSSI), which suffers from multipath interference in complicated indoor environments. Through the in-depth analysis of the multipath interference, an RSSI-assisted time difference of arrival (TDoA) method is proposed for Wi-Fi-based indoor position sensing in this work. The key idea is to compensate for the multipath interference in the received signals based on the coarse estimation using RSSI and TDoA calculation. A prototype system has been implemented to validate the proposed method. Experimental results have demonstrated the effectiveness of the proposed method, especially for handling the multipath interference with small propagation delay difference. Experimental results show that the indoor position sensing system can achieve a 90th percentile error of 0.3 m. The proposed method can also achieve moderate computational complexity and moderate real-time performance compared to other methods.

## 1. Introduction

In recent years, with the rapid development of personal mobile devices, the location based service has become increasingly indispensable in the daily life. The global position system (GPS) [1] has been widely used in the outdoor environment for positioning and navigation. However, the satellite signal is heavily attenuated inside buildings, making the GPS incapable of indoor position sensing. Since many people spend the vast majority of time indoors, it is important to develop real-time indoor position technologies which require minimal prior knowledge of the surroundings.

**Figure 1:**
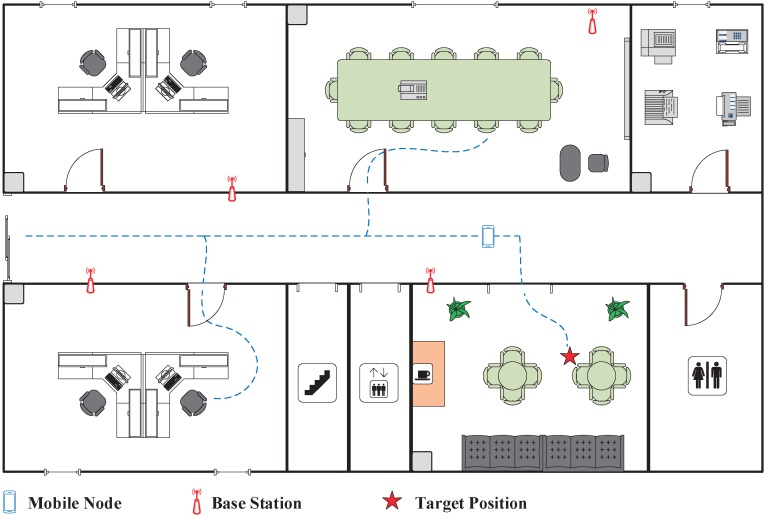
The Wi-Fi-based indoor position sensing.

The demands of indoor position sensing arise in various applications, such as human guiding [2], public security [3], classified advertisements [4], valuables monitoring [5], emergency rescue [6], underground parking [7] and augmented reality [8]. Researchers have investigated a wide variety of wireless signals with different physical properties or protocols for indoor position sensing, including infrared signals [9], ultrasound signals [10], and radio signals such as the Bluetooth [11], radio frequency identification (RFID) signals [12], ultra-wideband (UWB) signals [13], and Wi-Fi signals. Among those techniques, the Wi-Fi-based indoor position sensing has the advantages of low infrastructure cost and high reliability, since Wi-Fi networks are now extensively accessible in the residential areas, office spaces, and commercial districts. As shown in Figure 1, the Wi-Fi-based position sensing solution can provide the seamless positioning service in most of the current indoor environments using the existing Wi-Fi access points (APs).

The fundamental principle of Wi-Fi-based indoor position sensing is conducted in three phases. Firstly, Wi-Fi frames between the targeted mobile device and the APs (i.e., base stations) are collected as the raw data. Secondly, the key feature is extracted from the raw data which contains the position information. Finally, the feature information is processed using the position calculation algorithms to estimate the position of the targeted mobile device. Unlike the line-of-sight (LoS) signal transmission path in a GPS, indoor position sensing is confronted with complicated communication channel situations. The key challenges include multipath propagation, fading, noise and the environmental dynamics [14].

A number of Wi-Fi-based indoor position sensing methods have been reported in the literature. Roughly, those methods can be classified into two categories, namely, the received signal feature-based methods, and the propagation time-based methods [15,16,17,18,19,20,21,22,23,24,25,26,27,28,29,30,31,32,33,34,35,36,37,38]. The received signal strength indicator (RSSI) fingerprint is the most widely adopted feature to indicate position information in favor of its simplicity and accessibility [15,16,17,18,19,20,21,22,23,24,25]. Generally, the fingerprint based schemes are conducted in two phases, i.e., an offline phase followed by an online phase. In the offline phase, the RSSI vector from all the detectable APs is collected at a reference point (RP). By traversing all RPs in the object region, the fingerprint database is established. After the site survey, the position information is featured by the signal pattern. At a certain RP, the RSSI values are distinguished by the AP names or media access control (MAC) address. Thus, it is not required to know the exact positions of all APs, which leads to the advantage of deployment flexibility. In the online phase, the target position is estimated by comparing the current RSSI vector to the fingerprint database using the matching algorithms such as the maximum likelihood [15], k-nearest neighbors [16], support vector machine [17], random forest [18], Bayesian network [19], Gaussian process [20] and an artificial neural network [21]. The key system parameters, such as the number of APs, the density of RPs and the time span of signal collection determine the positioning precision. Duc V. Le et al. [22] used a deep belief network to train an unsupervised deep feature learning model, and this method helped to reduce the number of labeled fingerprints. In most fingerprint schemes, the RSSI values are extracted from the MAC layer. In contrast, [23,24] acquired the channel state information (CSI) from the physical layer (PHY), which is fine-grained for the channel characterization. Wang et al. [25] collected the amplitude and phase of 30 subcarriers from three antennas to establish the fingerprint database. Bisio et al. [26,27] proposed a probabilistic fingerprinting method which helped to reduce the computational complexity and energy consumption of the RSSI-based methods. Nevertheless, the offline phase in the received signal feature-based methods is laborious and time-consuming, and the positioning precision is prone to the surrounding changes.

The time of arrival (ToA) and the time difference of arrival (TDoA) methods are the commonly used propagation time-based methods [28,29,30,31,32,33,34,35,36,37,38]. The signal propagation time or propagation time difference between the mobile device and the APs is extracted from the received signals by the APs. Afterwards, the position calculating algorithm converts time information to range information. In the ToA methods [28,29] the signal transmitter (the mobile device) and all receivers (APs) are required to be strictly synchronized with nanosecond accuracy. In the TDoA methods [30,31,32,33,34,35,36,37,38], the propagation time difference for different receivers is measured, and then the transmitter position is calculated. The time synchronization is required among the receivers in the TDoA systems. The conventional position calculation algorithms include the direct solving method [30], the non-iterative maximum likelihood estimation method [31] and the Taylor-series method [32]. König et al. [33] calculated the TDoA by cross-correlating the received signal with a time continuous Barker code sequence. Exel et al. [34] designed synchronized receivers to precisely obtain the arrival time of the predefined timestamp. The super-resolution technique (SRT) was also adopted to estimate the non-LoS components, such as the ESPRIT [35], MUSIC [36], and MinNorm [37] methods, which separate the signal subspace from the noise subspace with the eigenvalue decomposition of covariance matrix. To reduce the computational burden, the Matrix Pencil (MP) algorithm was introduced [38]. To further improve the performance of the ToA or TDoA methods in a real indoor environment, extra attention needs to be paid to the multipath interference effect which largely downgrades position sensing accuracy.

In this work, a Wi-Fi-based indoor position sensing method with multipath interference mitigation is proposed. The major accomplishments of this work include:

1. The mechanism of the position sensing accuracy loss due to the multipath interference effect is analyzed theoretically, and the multipath strength indicator is defined to measure the interference quantitatively.
2. A novel RSSI-assisted TDoA method is proposed to mitigate the impact of the multipath interference. Especially, the proposed method is capable of handling the circumstances with small propagation delay difference.
3. The prototype of an RSSI-assisted TDoA position sensing (RTPS) system has been implemented in a software defined radio (SDR) platform. The prototype system shows advantages of high accuracy, high robustness, and low computational complexity compared to other methods in the literature.

This work gives a new method to fuse the classical RSSI and TDoA methods for the performance improvement, with the cost of extra signal processing steps compared to the classical methods. Unlike the TDoA methods in the literature, the proposed method can be used to handle the multipath effect with very small propagation delay difference between the LoS and non-LoS signals. The key contribution of this work is to use the RSSI result to coarsely estimate the multipath interference, and then to use the coarse estimation information to improve the TDoA measurement performance. To the best of our knowledge, there is no similar method reported by other research groups that combines both methods in such a way.

The rest of this paper is organized as follows. Section 2 investigates the effect of multipath interference, and gives details of the proposed RSSI-assisted TDoA method to solve this problem. Section 3 introduces the RTPS prototype structure and signal processing flow. The experimental setup and results are given in Section 4. The work is summarized in Section 5.

## 2. RSSI-Assisted TDoA Method with Multipath Interference Mitigation

The proposed indoor position sensing system is based on the IEEE 802.11b [39] Wi-Fi protocol, and it can also be applied to other wireless local area network (WLAN) protocols. The IEEE 802.11b system is a spread spectrum system. At the transmitter (mobile node) side, a pseudo-noise (PN) sequence is adopted to spread the spectrum of the baseband signal. The TDoA values are calculated by letting the mobile node send a frame repeatedly, and then cross-correlating the signals received by the base stations.

**Figure 2:**
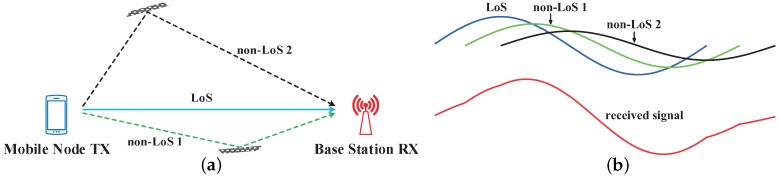
Multipath propagation: (**a**) Signal propagation with the LoS path and two non-LoS paths; (**b**) Actual received signal is a combination of LoS signal and non-LoS signals.

There are a few factors that affect the TDoA estimation accuracy. This work focuses on the non-ideality caused by the multipath interference. Figure 2a shows an example of the multipath propagation with one LoS path and two non-LoS paths in an indoor environment. In the non-LoS paths, the signals from the transmitter are reflected by the obstacles (e.g., the wall, the floor and the furniture), and propagate in multiple directions with different fading and delay. Compared to the LoS signal, the non-LoS signals propagate through longer distances with more path loss. The received signal at the base station side is the summation of the LoS and non-LoS signals, as shown in Figure 2b.

It can be shown that the accuracy of the TDoA calculation is greatly affected by the multipath interference. The proposed RSSI-assisted TDoA method is introduced to mitigate this problem. In this section, the conventional TDoA method will be introduced first, and then the proposed RSSI-assisted TDoA method will be described in details.

Although the target of this work is to provide a 2D position sensing method, the basic idea will be explained for the 1D position sensing case in which the mobile node moves along the line connecting two base stations. The presented method for a 1D case can be easily expanded to the 2D case.

### 2.1. Conventional TDoA Method for Position Sensing

To focus on the multipath effect, the non-ideal factors such as the frequency offset and the noise interference are not taken into consideration in this analysis. According to the IEEE 802.11b protocol, at the transmitter of the mobile node, the direct sequence spread spectrum (DSSS) PHY with the Barker code $\left\{d_{n}\right\}=\left\{+1,-1,+1,+1,-1,+1,+1,+1,-1,-1,-1\right\}$ is used to spread the spectrum of the modulated signals. The transmitted baseband signal $s\left(t\right)$ is represented by

(1) $$s\left(t\right)=\sum_{n}d_{n}\cdot r\left(t-nT_{c}\right)$$

where $\left\{d_{n}\right\}$ is the transmitted sequence after DSS, $r\left(t\right)$ is the raised cosine function, and $1/T_{c}=11$ Mbps is the chirp rate. The time domain channel impulse response (CIR) with the multipath propagation is modeled as

(2) $$h\left(t\right)=\sum_{i=0}^{N}a_{i}\cdot \delta \left(t-\tau_{i}\right)$$

in which *N* is the number of non-LoS channels, $a_{i}=\left|a_{i}\right|e^{j\theta_{i}}$ defines the complex attenuation of each propagation channel including the LoS channel $Ch_{0}$ and the non-LoS channels $Ch_{1}$, ..., $Ch_{N}$, and $\tau_{i}$ denotes the propagation time of the $i_{th}$ channel (i = 0,1, ..., N). To represent the situation with two base stations, two CIRs are differentiated by additional subscripts.

(3) $$\left\{\begin{array}{c} h_{1}\left(t\right)=\sum_{i=0}^{N_{1}}a_{1,i}\cdot \delta \left(t-\tau_{1,i}\right)\\ h_{2}\left(t\right)=\sum_{i=0}^{N_{2}}a_{2,i}\cdot \delta \left(t-\tau_{2,i}\right) \end{array}\right.$$

For simplicity, it is assumed that $\tau_{1,0}=0$, and let $\Delta t=\tau_{2,0}-\tau_{1,0}$. With the electromagnetic wave propagation speed known as *c*, Δt actually represents the position information which needs to be solved, if the mobile node moves between the two base stations and the distance between the two base stations *L* is fixed. For example, when Δt=0, the position of the transmitter is in the middle of the two base stations. When Δt>0, the transmitter is more closer to the first base station, the distance to the first base station is calculated as L/2−cΔt/2.

At the base station sides, the baseband signals received by the two receivers are described as

(4) $$\left\{\begin{array}{c} \begin{array}{cc} y_{1}\left(t\right) & =s\left(t\right)*h_{1}\left(t\right)=a_{1,0}s\left(t\right)+\sum_{i=1}^{N_{1}}a_{1,i}cos\left(\omega_{c}\Delta \tau_{1,i}\right)s\left(t-\Delta \tau_{1,i}\right)\\ y_{2}\left(t\right) & =s\left(t\right)*h_{2}\left(t\right)=a_{2,0}s\left(t+\Delta t\right)+\sum_{i=1}^{N_{2}}a_{2,i}cos\left(\omega_{c}\Delta \tau_{2,i}\right)s\left(t+\Delta t-\Delta \tau_{2,i}\right) \end{array} \end{array}\right.$$

in which $\omega_{c}$ is the transceiver carrier frequency, $\Delta \tau_{1,i}=\tau_{1,i}-\tau_{1,0}$ is the propagation delay difference between the LoS path and the $i_{th}$ non-LoS path, and so is $\Delta \tau_{2,i}$.

First consider the situation that there is no multipath effect, and the two received signals are simplified to

(5) $$\left\{\begin{array}{c} \begin{array}{cc} y_{1}\left(t\right) & =a_{1,0}s\left(t\right)\\ y_{2}\left(t\right) & =a_{2,0}s\left(t+\Delta t\right) \end{array} \end{array}\right.$$

The received signals are quantized to two sequences $y_{1}\left(nT_{s}\right)$ and $y_{2}\left(nT_{s}\right)$, using the baseband ADCs with a sampling rate of 88 Msps. The ADC sampling clock cycle period is denoted as $T_{s}$. Denote the cross-correlation between $y_{1}\left(t-\tau \right)$ and $y_{2}\left(t\right)$ as $R\left(\tau \right)=E\left[y_{1}\left(t-\tau \right)y_{2}\left(t\right)\right]$. The actual calculated cross-correlation is discrete, and denoted as $R_{i}\;\left(i=0,\pm 1,\pm 2,\ldots \right)=R\left(iT_{s}\right)$. $R_{i}$ with *N* elements from $y_{1}\left(nT_{s}\right)$ and $y_{2}\left(nT_{s}\right)$ is calculated as

(6) $$R_{i}=\sum_{n=1}^{N}y_{1}\left(nT_{s}-iT_{s}\right)y_{2}\left(nT_{s}\right),\;i=-N/2+1,\ldots ,0,\ldots ,N/2$$

**Figure 3:**
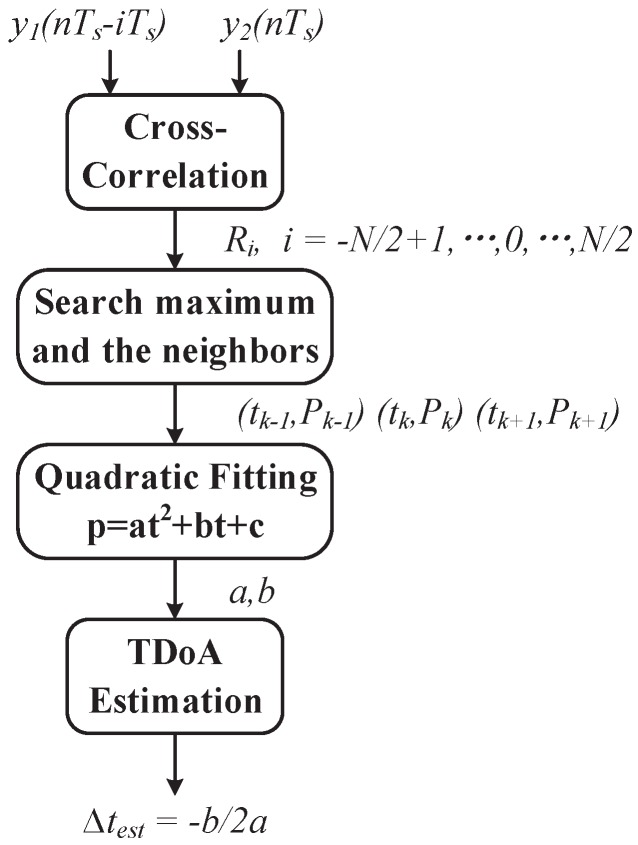
TDoA estimation based on cross-correlation calculation and quadratic fitting.

The processing flow of TDoA calculation is shown in Figure 3. The maximum $R_{i}$ is found as $P_{k}$ with the corresponding time shift $t_{k}$. Roughly, $t_{k}$ can be used to approximate Δt, and the accuracy is actually limited by the time resolution $T_{s}$. To achieve a fine resolution, the quadratic fitting method [40] is used. In the quadratic fitting, the cross-correlation curve is viewed as a convex parabola in the neighborhood of discrete peak point $\left(t_{k},P_{k}\right)$, and three discrete points $\left(t_{k-1},P_{k-1}\right)$, $\left(t_{k},P_{k}\right)$ and $\left(t_{k+1},P_{k+1}\right)$ are used to perform the fitting. Note that $P_{k}=R\left(t_{k}\right)$, $t_{k-1}=t_{k}-T_{s}$, and $t_{k+1}=t_{k}+T_{s}$. The quadratic equation coefficients ${\left[a,b,c\right]}^{T}$ are calculated by

(7) $$\begin{array}{c} \left[\begin{array}{c} a\\ b\\ c \end{array}\right]={\left[\begin{array}{ccc} t_{k-1}^{2} & t_{k-1} & 1\\ t_{k}^{2} & t_{k} & 1\\ t_{k+1}^{2} & t_{k+1} & 1 \end{array}\right]}^{-1}\left[\begin{array}{c} P_{k-1}\\ P_{k}\\ P_{k+1} \end{array}\right] \end{array}$$

The time shift of the peak point on the fitted parabola is given by $-\frac{b}{2a}$, which can be used as an estimation of Δt, i.e.,

(8) $$\Delta t_{est}=-\frac{b}{2a}$$

The distance to the base station is calculated as

(9) $$\left\{\begin{array}{c} \begin{array}{cc} d_{1est\_t} & =\frac{L}{2}-\frac{c\Delta t_{est}}{2}\\ d_{2est\_t} & =\frac{L}{2}+\frac{c\Delta t_{est}}{2} \end{array} \end{array}\right.$$

**Figure 4:**
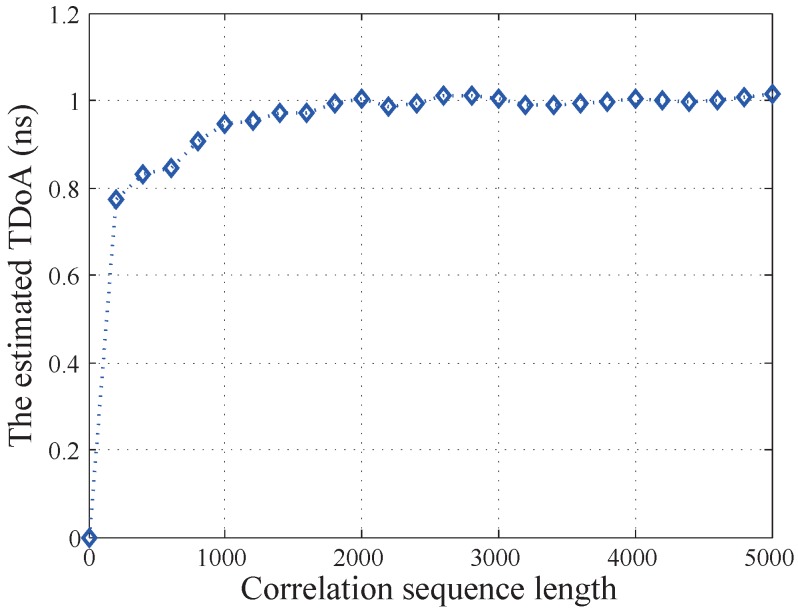
Estimated TDoA vs. correlation sequence length *N* (1 ns TDoA).

As proved in [41], if there is only the white noise in the channel, the quadratic fitting method gives an estimation error variance calculated as

(10) $$\sigma^{2}=\frac{1}{N}\frac{3}{4\pi^{2}W^{2}}\left(\frac{1+2SNR}{SNR^{2}}+1\right)$$

in which *N* is the length of correlation sequence, *W* is the noise bandwidth of white noise, and SNR is received signal-noise ratio. To find an appropriate *N*, the numerical simulation has been performed. Figure 4 shows the estimated TDoA versus different *N*. In this simulation, the actual TDoA is 1 ns, and the SNR is set to 15 dB. Based on Figure 4, the correlation length *N* is chosen to be 2200.

### 2.2. Proposed RSSI-Assisted TDoA Method

Now consider the situation with a multipath effect. There may exist multiple non-LoS channels in a certain environment. Note that the non-LoS signals fade quickly due to the reflection/refraction loss and the longer propagation paths compared to the LoS path. For a given short time interval in which the RSSI and TDoA are measured, usually only one non-LoS path needs to be considered, which may give the signal strength comparable to that of the LoS path. Without loss of generality, in the following analysis only the major non-LoS component is included in the received signal $y_{1}\left(t\right)$ and $y_{2}\left(t\right)$.

Thus, Equation (4) is simplified to

(11) $$\left\{\begin{array}{c} \begin{array}{cc} y_{1}\left(t\right) & =a_{1,0}s\left(t\right)+a_{1,1}cos\left(\omega_{c}\Delta \tau_{1,1}\right)s\left(t-\Delta \tau_{1,1}\right)\\ y_{2}\left(t\right) & =a_{2,0}s\left(t+\Delta t\right)+a_{2,1}cos\left(\omega_{c}\Delta \tau_{2,1}\right)s\left(t+\Delta t-\Delta \tau_{2,1}\right) \end{array} \end{array}\right.$$

Rewrite Equation (11) as

(12) $$\left\{\begin{array}{c} \begin{array}{cc} y_{1}\left(t\right) & =a_{1,0}\left[s\left(t\right)+\frac{a_{1,1}}{a_{1,0}}cos\left(\omega_{c}\Delta \tau_{1,1}\right)s\left(t-\Delta \tau_{1,1}\right)\right]\\ y_{2}\left(t\right) & =a_{2,0}\left[s\left(t+\Delta t\right)+\frac{a_{2,1}}{a_{2,0}}cos\left(\omega_{c}\Delta \tau_{2,1}\right)s\left(t+\Delta t-\Delta \tau_{2,1}\right)\right] \end{array} \end{array}\right.$$

Now define the multipath strength indicator (MPSI) as follows, which can be used to measure the impact of multipath interference.

(13) $$MPSI_{i}=\frac{a_{i,1}}{a_{i,0}}cos\left(\omega_{c}\Delta \tau_{i,1}\right),\;i=1,2$$

Since the non-LoS path propagation distance is larger than that of the LoS path, it is expected $\left|\frac{a_{i,1}}{a_{i,0}}\right|$ is less than 1, and $\left|MPSI_{i}\right|$ is also less than 1. If MPSI>0, the total signal strength received by the base station will be larger than that through the LoS path only, and vice versa.

After normalization, Equation (12) can be written as

(14) $$\left\{\begin{array}{c} \begin{array}{cc} y_{1}\left(t\right) & =s\left(t\right)+MPSI_{1}s\left(t-\Delta \tau_{1,1}\right)\\ y_{2}\left(t\right) & =s\left(t+\Delta t\right)+MPSI_{2}s\left(t+\Delta t-\Delta \tau_{2,1}\right) \end{array} \end{array}\right.$$

With the multipath interference present, the calculated peak cross-correlation and its neighbors, i.e., $P_{k-1}^{\prime }$, $P_{k}^{\prime }$ and $P_{k+1}^{\prime }$, are different from those without the multipath interference denoted as $P_{k-1}$, $P_{k}$ and $P_{k+1}$.

(15) $$\left\{\begin{array}{c} \begin{array}{cc} P_{k-1}^{\prime }=P_{k-1} & +MPSI_{1}R\left(T_{s}+\Delta t+\Delta \tau_{1,1}\right)+MPSI_{2}R\left(T_{s}+\Delta t-\Delta \tau_{2,1}\right)\\ & +MPSI_{1}MPSI_{2}R\left(T_{s}+\Delta t+\Delta \tau_{1,1}-\Delta \tau_{2,1}\right)\\ P_{k}^{\prime }=P_{k} & +MPSI_{1}R\left(\Delta t+\Delta \tau_{1,1}\right)+MPSI_{2}R\left(\Delta t-\Delta \tau_{2,1}\right)\\ & +MPSI_{1}MPSI_{2}R\left(\Delta t+\Delta \tau_{1,1}-\Delta \tau_{2,1}\right)\\ P_{k+1}^{\prime }=P_{k+1} & +MPSI_{1}R\left(T_{s}-\Delta t-\Delta \tau_{1,1}\right)+MPSI_{2}R\left(T_{s}-\Delta t+\Delta \tau_{2,1}\right)\\ & +MPSI_{1}MPSI_{2}R\left(T_{s}-\Delta t-\Delta \tau_{1,1}+\Delta \tau_{2,1}\right) \end{array} \end{array}\right.$$

**Figure 5:**
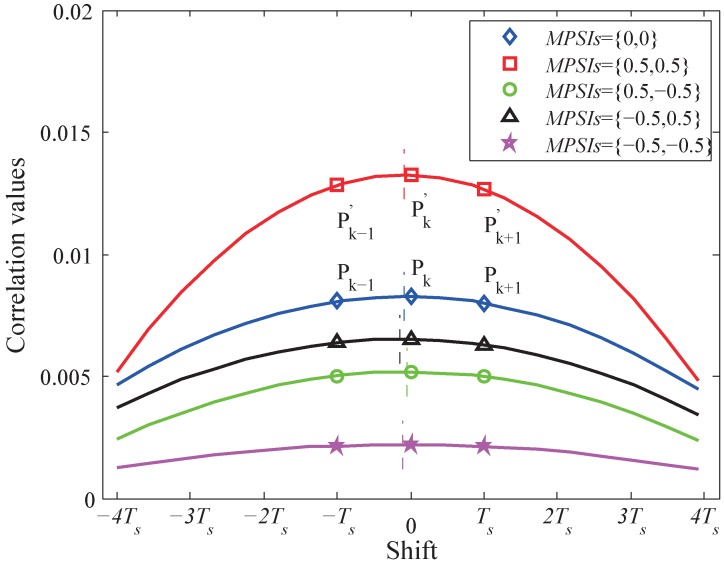
Cross-correlation values under different multipath combinations (Δt = −1 ns): the vertical line on each curve represents the estimated TDoA $\Delta t_{est}$.

Obviously, the additional terms added to $P_{k-1}$, $P_{k}$ and $P_{k+1}$ will cause error to the TDoA estimation using the aforementioned quadratic fitting method. It has been observed and then verified through the numerical simulation that only a negative MPSI will introduce large estimation error. Figure 5 shows how different $MPSI_{1}$ and $MPSI_{2}$ combinations affect the TDoA estimation result. If both $MPSI_{1}$ and $MPSI_{2}$ are positive (less than 0.5), $P_{k-1}^{\prime }$, $P_{k}^{\prime }$ and $P_{k+1}^{\prime }$ are just vertically shifted upward compared to $P_{k-1}$, $P_{k}$ and $P_{k+1}$, the peak estimation using quadratic fitting is barely affected. On the other hand, if either $MPSI_{1}$ or $MPSI_{2}$ is negative, the vertical shifts between $P_{k-1}^{\prime }$, $P_{k}^{\prime }$, $P_{k+1}^{\prime }$ and $P_{k-1}$, $P_{k}$, $P_{k+1}$ are not balanced, and the quadratic fitting will leads to a horizontally shifted peak position estimation, in other words, a $\Delta t_{est}$ with some error compared to Δt.

The above phenomenon suggests that if the negative non-LoS component in $y_{1}\left(t\right)$ and $y_{2}\left(t\right)$ can be recognized and compensated for, the estimation error due to the multipath interference can be mitigated. Also note that the TDoA estimation is quite robust for positive MPSI up to 0.5 which is validated by various numerical simulations; this compensation can be accomplished by roughly adding a compensation signal $y_{c}\left(t\right)$ with positive and relatively large MPSI such as 0.5 to the received $y_{1}\left(t\right)$ or $y_{2}\left(t\right)$. $y_{c}\left(t\right)$ can be constructed as follows

(16) $$y_{c}\left(t\right)=a_{c}s\left(t-\tau_{c}\right)$$

in which $a_{c}$ is chosen to make the amplitude of $y_{c}\left(t\right)$ is roughly 0.5 times that of $y\left(t\right)$ which is compensated. An empirical method to obtain the appropriate $\tau_{c}$ is to find $\tau_{c}$ that minimizes $\sum_{t}^{}\left|s\left(t-\tau_{c}\right)-y\left(t\right)\right|$, and this method has been verified through both numerical simulations and experiments. After the compensation, $y_{1}^{\prime }\left(t\right)=y_{1}\left(t\right)+y_{c1}\left(t\right)$ and $y_{2}^{\prime }\left(t\right)=y_{2}\left(t\right)+y_{c2}\left(t\right)$ are processed using the TDoA method described in previous part of this section to obtain the fine position estimation. The remaining question is then how to recognize the polarity of MPSI in the received signals.

In this work, the received signal strength indicator (RSSI) is used to recognize the polarity of the MPSI. For each base station, the RSSI is calculated using N=2200 sampling points. The distance *d* between the mobile node and the base station is then obtained using the log-normal shadowing model [42] described by

(17) $$RSSI=RSSI_{0}-10nlog_{10}\frac{d}{d_{0}}$$

in which RSSI is the received signal strength indicator (in dB) by the base station at the distance *d* from the transmitter, $d_{0}$ is the reference distance, $RSSI_{0}$ is the signal strength at the reference distance, and *n* is the path loss exponent. $RSSI_{0}$ and *n* are fixed for a system with given transmitted signal power, antennas and $d_{0}$, and can be measured for a real system. The estimated distance $d_{est\_rssi}$ is calculated as

(18) $$d_{est\_rssi}=d_{0}10^{\frac{RSSI_{0}-RSSI}{10n}}$$

For the system with two base stations, the estimated distances between the mobile node and the two base stations based on the RSSI are denoted as $d_{1est\_rssi}$ and $d_{2est\_rssi}$, respectively. If there is no multipath interference or any other non-ideal factor, it is expected that the summation of $d_{1est\_rssi}$ and $d_{2est\_rssi}$ is exactly the distance *L* between the two base stations, when the mobile node moves between the base stations. If $d_{1est\_rssi}+d_{2est\_rssi}>L$, it can be inferred that the received signal is attenuated due to the negative MPSI, which makes the estimated distance larger than the true value. In this work, the following procedure is used to determine the polarity of $MPSI_{1}$ and $MPSI_{2}$, and implement the compensation.

1. If $d_{1est\_rssi}+d_{2est\_rssi}>\left(1+\alpha \right)L$, it is surmised that both $MPSI_{1}$ and $MPSI_{2}$ are negative, and the compensation signal $y_{c1}\left(t\right)$ and $y_{c2}\left(t\right)$ will be found and added to $y_{1}\left(t\right)$ and $y_{2}\left(t\right)$, respectively.
2. If $\left(1+\beta \right)L<d_{1est\_rssi}+d_{2est\_rssi}<\left(1+\alpha \right)L$, it is surmised that either $MPSI_{1}$ or $MPSI_{2}$ is negative, and the compensation will be applied to the one selected from $y_{1}\left(t\right)$ or $y_{2}\left(t\right)$ which has larger $d_{est\_rssi}$.
3. If $L<d_{1est\_rssi}+d_{2est\_rssi}<\left(1+\beta \right)L$ and there is large difference between $\Delta d_{est\_rssi}=d_{2est\_rssi}-d_{1est\_rssi}$ and $\Delta d_{est\_t}=d_{2est\_t}-d_{1est\_t}$ (specifically two conditions: i. the polarity of $\Delta d_{est\_rssi}$ is different from that of $\Delta d_{est\_t}$; ii. $\Delta d_{est\_rssi}$ and $\Delta d_{est\_t}$ have the same polarities, but $\Delta d_{est\_rssi}$/$\Delta d_{est\_t}$ is less than a threshold, i.e., 0.1), the compensation will be applied to the one selected from $y_{1}\left(t\right)$ or $y_{2}\left(t\right)$ which has larger $d_{est\_rssi}$.
4. No compensation will be applied for all the other conditions.

The values of α and β can be found and optimized through simulations and experiments. In this work, α and β are chosen to 1.4 and 1.1, respectively, for the 1D position sensing case.

**Figure 6:**
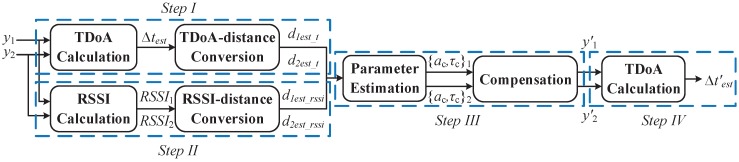
Processing flowchart of the proposed RSSI-assisted TDoA method.

In summary, the proposed RSSI-assisted TDoA method for the 1D position sensing is composed of the following steps, which is also shown in Figure 6. Note that only the polarity of the MPSI instead of the non-LoS component itself is estimated based on the RSSI. The compensation is only used to change the non-LoS component polarity, but not to precisely eliminate the non-LoS component.

*Step I:* Use the cross-correlation calculation and the quadratic fitting to coarsely estimate the mobile node position $d_{1est\_t}$ and $d_{2est\_t}$;

*Step II:* Use the RSSI to coarsely estimate the mobile node position $d_{1est\_rssi}$ and $d_{2est\_rssi}$;

*Step III:* Based on the coarse estimation in two preceding steps, determine the polarity of the multipath interference in the received signals, and compensate for the multipath interference in the signals $y_{1}\left(t\right)$ and/or $y_{2}\left(t\right)$ received by the base stations;

*Step IV:* Repeat Step I by using the signals after compensation $y_{1}^{\prime }\left(t\right)$ and $y_{2}^{\prime }\left(t\right)$ to obtain the fine estimation of the mobile node position.

The same method is used for the 2D position sensing, and the detailed processing procedure will be explained in Section 4.

## 3. Prototype System for Method Validation

To validate the proposed RSSI-assisted TDoA method, a prototype system has been implemented. In this section, the hardware and signal processing implementation of the proposed TDoA position sensing method are presented.

### 3.1. Prototype System Hardware and Firmware

**Figure 7:**
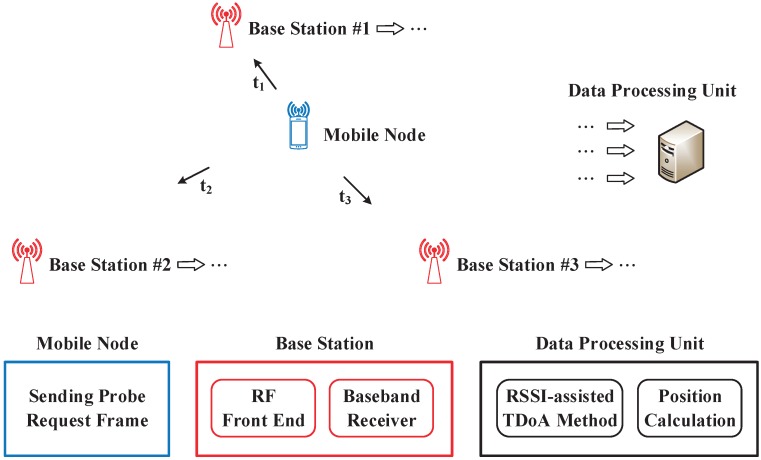
Overall system architecture.

The overall architecture of the proposed indoor position sensing system is given in Figure 7, which is composed of one mobile node, two or three base stations depending on the 1D or 2D sensing, and a data processing unit. Both the mobile node and the base stations are implemented using the NI USRP software defined radio devices. The mobile node automatically sends the positioning request by broadcasting the probe request frame periodically. In the base stations, a dedicated baseband receiver following the RF front-end is employed to mitigate the carrier frequency offset and improve the received SNR. The proposed RSSI-assisted TDoA algorithm is implemented in the PC based data processing unit that is connected to the base stations using cables.

**Figure 8:**
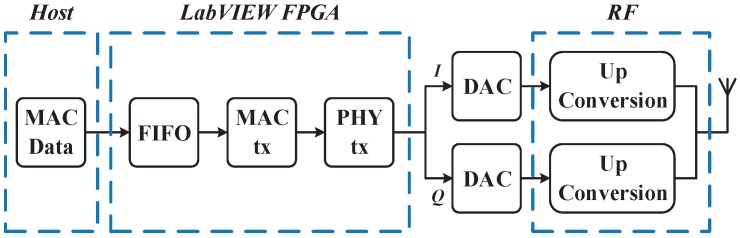
Functional diagram of the mobile node.

The mobile node is implemented using an NI USRP-2920 device, and the related functional blocks are shown in Figure 8. For the scenario where the mobile node sends a positioning request, the probe request frame is selected as the request signal. The probe request frame is in the category of the management frame, strictly in compliance with the IEEE 802.11b protocol. The MAC service data unit (MSDU) is implemented in the host PC and sent to the baseband FPGA in the USRP-2920 device through FIFO. In the MAC layer implemented in the FPGA, the MAC protocol data unit (MPDU) is generated by joining the MAC header to MSDU. In the PHY layer, the MPDU is prefixed with the physical layer convergence protocol (PLCP) preamble and header. Then, the In-phase (I) and Quadrature-phase (Q) signals are processed in turn by the scrambler, the modulator, the DSSS and the pulse shaping filter. The I/Q baseband signals are then converted to the analog signals using the DACs, and up-converted to the 2.4 GHz frequency band.

**Figure 9:**
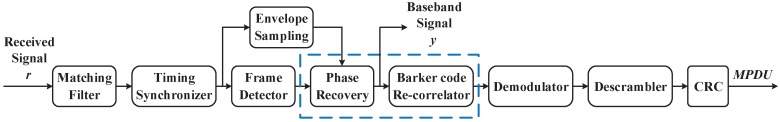
The architecture of baseband receiver.

The base stations are implemented using NI USRP-2952R devices. Each USRP-2952R device is composed of two receiving channels, and each channel serves as a base station. Therefore, two USRP-2952R devices are needed to implement three base stations. Note that all the base stations need to be synchronized. The two receiving channels within each single device is inherently strictly synchronized. Two USRP-2952R devices are synchronized by deploying the master–slave mode. The slave USRP-2952R device is synchronized to the master device by receiving a time reference signal from the master through a coaxial cable. Three base stations are identical, consisting of the RF front end, the ADCs and the baseband receiver. The baseband receiver processes the received signal *r* to generate the baseband signal *y* for the subsequent protocol parsing and the proposed RSSI-assisted TDoA method. The architecture of the baseband receiver in the base station is depicted in Figure 9.

The phase recovery block is specially designed to compensate for the carrier frequency offset between the mobile node transmitter and the base station receivers. The frequency offset results in an additional envelope in the received signal, and will finally cause the drift in the cross-correlation calculation. The classic frequency offset correction algorithms are based on the Fast Fourier Transform (FFT), which requires a large number of data points. In this work, the Coordinate Rotation Digital Computer (CORDIC) algorithm proposed in [43] is adopted to solve the frequency offset issue. The received signal is described as

(19) $$r\left(n\right)=s\left(n\right)\cdot exp\left[j\left(2\pi \Delta fnT_{s}+\Delta \varphi \right)\right]$$

in which $s\left(n\right)$ represents the sampled version of the transmitted baseband signal given in Equation (1), Δf is the frequency offset, Δφ is the phase offset, and $T_{s}$ is the ADC sampling period at the receiver side. The conjugate multiplication *z* of two sampling points spaced by *N* is expressed as follows

(20) $$\begin{array}{cc} z & =r\left(n\right)\cdot r^{*}\left(n+N\right)\\ & =s\left(n\right)\cdot s^{*}\left(n+N\right)\cdot exp\left(-j2\pi \Delta fnT_{s}\right)\\ & =\left|s\left(n\right)\right|\cdot \left|s\left(n+N\right)\right|\cdot exp\left[j\left(\varphi_{n}-\varphi_{n+N}\right)\right]\cdot exp\left(-j2\pi \Delta fnT_{s}\right) \end{array}$$

The envelope sampling block ensures that $r\left(n\right)$ and $r\left(n+N\right)$ are from the signal envelope. With DBPSK modulation, $\varphi_{n}-\varphi_{n+N}$ is either 0 or π. Therefore, Δf can be derived from

(21) $$\Delta f=\left|\frac{1}{2\pi NT_{s}}arctan\left(\frac{Im\left(z\right)}{Re\left(z\right)}\right)\right|$$

in which $Im\left(z\right)$ and $Re\left(z\right)$ are the imaginary part and real part of *z*. In the actual implementation, Δf is calculated and averaged based on eight sets of $\left\{r\left(n\right),r\left(n+N\right)\right\}$.

### 3.2. Signal Processing Flow for Position Sensing

**Figure 10:**
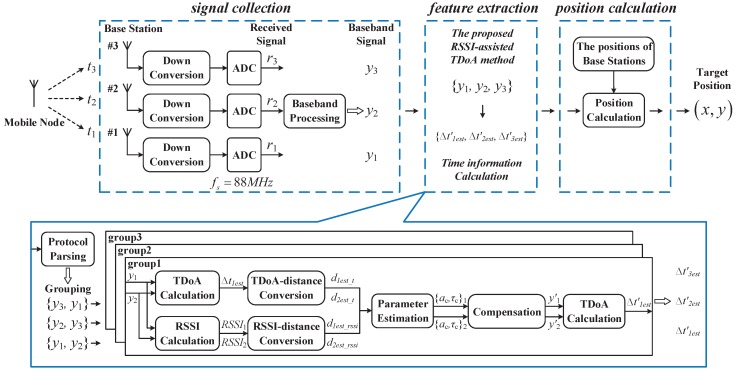
Signal processing flow for 2D position sensing.

The 1D position sensing is quite simple, and can be performed directly following the procedure described in Section 2. The overall data processing flow for 2D position sensing is shown in Figure 10. In the 2D position sensing case, the positioning request signal arrives at three base stations with the propagation time $t_{i}\;\left(i=1,2,3\right)$. At the base station side, the down-converted received signal $r_{i}$ is sampled by the 88 Msps ADCs. The baseband receiver calibrates the frequency offset and outputs the MPDU. The protocol parser labels the continuous arriving frames with the designated source address and timestamp. The baseband signals $y_{i}\;\left(i=1,2,3\right)$ from three base stations with the identical label are grouped in pairs as the input of the proposed RSSI-assisted TDoA method. Three time difference estimations, i.e., $\Delta t_{1est}^{\prime }$ between base station #1 and #2, $\Delta t_{2est}^{\prime }$ between base station #2 and #3, and $\Delta t_{3est}^{\prime }$ between base station #3 and #1, are obtained. The set of $\left\{\Delta t_{1est}^{\prime },\Delta t_{2est}^{\prime },\Delta t_{3est}^{\prime }\right\}$ is then used to calculate the 2D position with the predefined positions of base stations.

The conventional position calculating algorithms based on the TDoA include the Fang method [30], Chan method [31] and the Taylor-series method [32]. In the Taylor-series method [32], the iterative local least squares (LS) solution with an initial position guess is robust with high tolerance to the TDoA estimation errors, and this method is adopted for the position calculation in this work.

The presented method can be easily extended to the 3D positioning solution by modifying the processing flow in Figure 10: (1) increase the number of base stations (4 base station minimally); (2) calculate 6 TDoA values (the TDoA values between every two base stations) using the RSSI-assisted method in Section 2, if 4 base stations are used; (3) change the 2D coordinate calculation to the 3D coordinate calculation using the TDoA values.

## 4. Simulation and Experimental Results

The proposed RSSI-assisted TDoA method has been validated through both the numerical simulation and the experiments using the prototype system.

### 4.1. Simulation Results

In order to evaluate the algorithm’s performance, the numerical simulation is performed using MATLAB. The simulation of 1D position sensing with one mobile node and two base stations is described in this section. In this simulation, the mobile node is placed at a fixed position, and the theoretical TDoA is set to Δ*t* = −1 ns. The propagation channel between the mobile node and either base station consists of the LoS path and one non-LoS path with $MPSI\in \left[-0.5,0.5\right]$. Totally 49 combinations of $\left\{MPSI_{1},MPSI_{2}\right\}$ are simulated, and 5000 measurements with random phase noise in the received signal are calculated for each combination.

**Figure 11:**
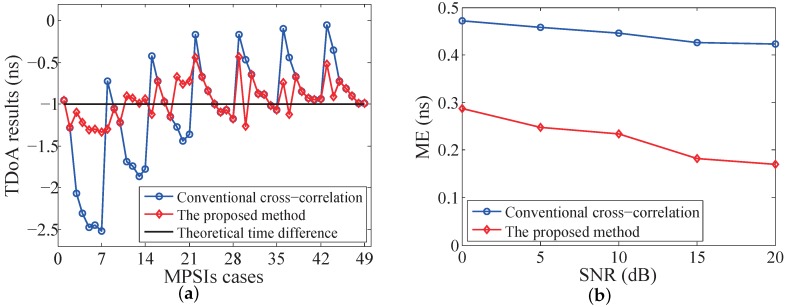
Simulation results of 1D TDoA estimation with different multipath and SNR conditions: (**a**) TDoA calculations of the conventional cross-correlation and the proposed method for 49 MPSIs cases and 15 dB SNR; (**b**) TDoA mean error for different received SNR.

Figure 11a shows the TDoA calculation results with the received SNR equal to 15 dB. In the simulation, the propagation delay difference is very small ($\Delta \tau \in \left[0.45\;\mathrm{ns},0.6\;\mathrm{ns}\right]$), which is far less than the ADC sampling period $T_{s}=1/88$ MHz = 11.36 ns. The conventional cross-correlation method gives a TDoA with quite a large error which can be up to 1.5 ns, and the error is correlated to the multipath interference. With the proposed method presented in Section 2, the maximum TDoA estimation error is reduced to only 0.5 ns. Figure 11b shows the mean error (ME) of TDoA estimation for different received SNR. The ME of the proposed method is about half of that by using the conventional cross-correlation method.

### 4.2. 1D Position Sensing Experiment

**Figure 12:**
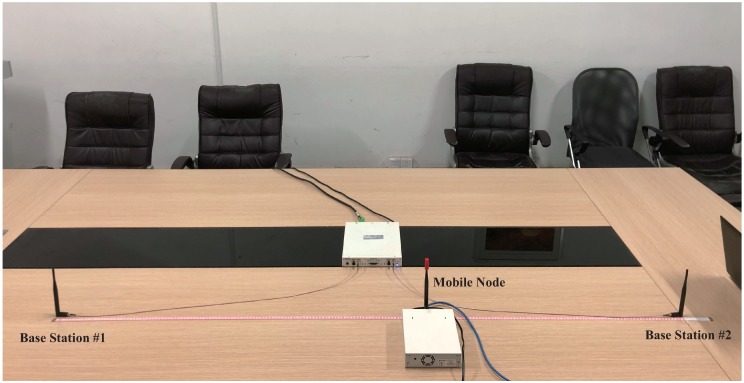
1D position sensing experimental setup to validate the proposed method.

To validate the proposed method, experiments on the prototype system are conducted in a meeting room. The 1D position sensing experiment setup with one mobile node and two base stations is shown in Figure 12. The receiving antennas of two base stations are arranged 2 m apart, and the mobile node moves along the straight line between two base stations. The mobile node broadcasts the probe request frame with a fixed interval of 10 ms. The probe request frame arrives at the receiving antennas via the LoS path and the non-LoS paths caused by the reflection by the desk surface, the surrounding chairs and walls. Seven test positions are uniformly distributed with 0.1 m space. The mobile node remains stationary at each test position for 5 s to collect adequate raw data.

**Figure 13:**
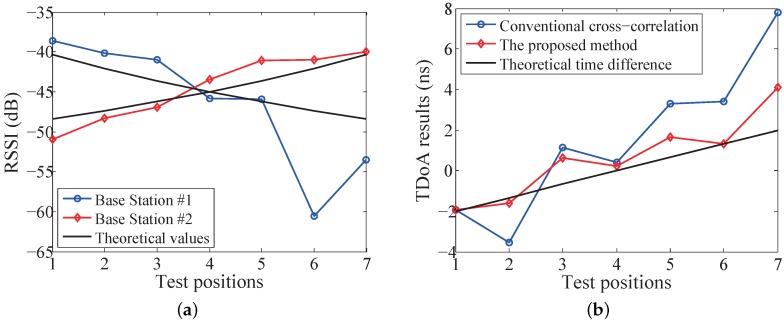
1D position sensing experimental results at 7 test positions: (**a**) RSSI values of base station #1 and #2; (**b**) TDoA calculation results of the conventional cross-correlation method and the proposed method.

The RSSI values of two base stations are shown in Figure 13a. The theoretical values are derived from the log-normal shadowing model as the reference. It can be found that the measured RSSI values deviate significantly from the theoretical values at position #6 and #7, which reflects the impact of the multipath interference. It is worth mentioning that small deviation does not represent the corresponding test position is immune to the multipath interference. The TDoA calculation results using the conventional cross-correlation and the proposed method are shown in Figure 13b. It is clear that the TDoA results calculated by the proposed method are closer to the theoretical prediction at all the seven positions. Lategahn et al. [44] presented a fusion method of RSSI and TDoA, which chose the relatively reliable result as the target position. However, it can be seen that the Lategahn method will not work well in this experiment setup, especially at position #6 and #7, for which neither the RSSI method nor the conventional TDoA method can give a good position estimation. In contrast, the proposed RSSI-assisted TDoA method gives a reliable position estimation, even at position #6 and #7.

### 4.3. 2D Position Sensing Experiment

**Figure 14:**
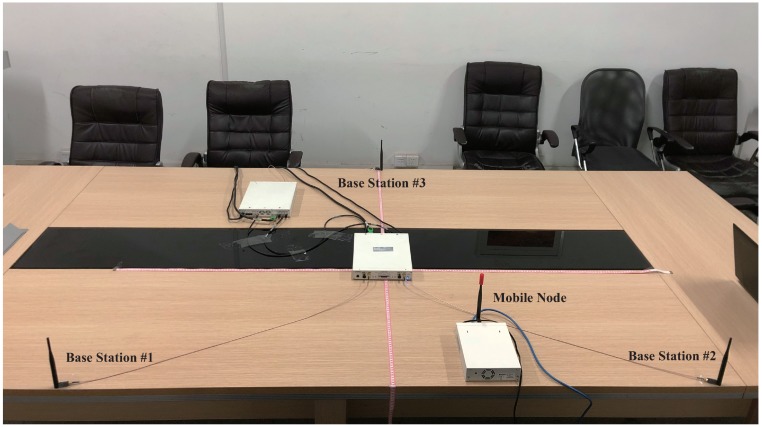
2D position sensing experimental setup to validate the proposed method.

The experimental setup for 2D position sensing is shown in Figure 14. It consists of one mobile node and three base stations. The mobile node moves on the surface of the table. Three base stations are strictly synchronized by sharing the clock source.

**Figure 15:**
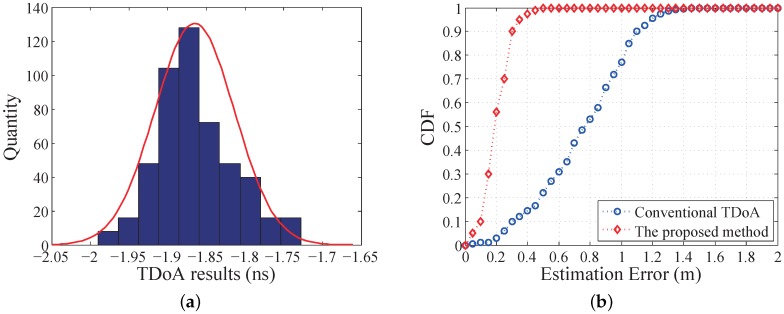
Experimental results of 2D position sensing: (**a**) Histogram of TDoA results distribution with the mobile node at a fixed test position; (**b**) Cumulative distribution function of estimation errors by using two methods.

Figure 15a shows the distribution histogram of the measured TDoA values between two base stations, by repeating the proposed method 500 times and the mobile node staying at one randomly selected position. At this position, the theoretical TDoA value is −1.33 ns. The measured TDoA result has a distribution close to a Gaussian distribution. The average measured TDoA is about −1.85 ns, and all the results are distributed in the range of ±0.15 ns. The maximum TDoA error is about 0.65 ns, which means that the maximum position estimation error is about 0.2 m. Figure 15b shows the cumulative distribution function (CDF) of the 2D position estimation errors using the proposed method and the conventional TDoA method. It is clearly seen that the proposed method gives a higher position estimation accuracy, with 90% of the measurement errors less than 0.3 m.

The performance of the presented RSSI-assisted TDoA method is summarized and compared to three representative works in the literature, as shown in Table 1. 90% of the position sensing errors in this work is less than 0.3 m. This result is actually better than that of the other works listed in Table 1. Note that [38] reported a root mean square (RMS) error of 0.23 m, and equivalently, the 90th percentile error would be more than 0.4 m. The proposed method gives the best position sensing accuracy. Compared to the other works in Table 1, the proposed method has also achieved moderate computational complexity and moderate real-time performance.

## 5. Conclusions

In this work, we analyzed how multipath interference affects indoor position sensing accuracy, and the multipath strength indicator is introduced to quantify the impact. An improved cross-correlation-based TDoA method is proposed by using RSSI calculation for coarse estimation. The main idea is to compensate for the multipath interference in the received signals with the RSSI values and the coarse TDoA results. To validate the effectiveness of the proposed method, a prototype system has been implemented in a software defined radio platform. Both the simulation and experimental results have demonstrated that the proposed method is effective to mitigate the multipath interference effect in position sensing with affordable computation overhead. Although the proposed method is validated based on the IEEE 802.11b Wi-Fi protocol, it can be easily transplanted to other wireless systems without any constraint on the signal type, channel bandwidth, MAC protocol, etc., which is valuable in IoT applications with self-organizing sensor networks.

Future work will probably focus on the verification of the proposed method in real application scenarios, by building an indoor position sensing system based on real mobile devices and off-the-shelf Wi-Fi access points. The proposed method will be extended to 3D position sensing situations with more base stations deployed in a 3D indoor space. Also, efforts will be made to verify the method on other wireless communication protocols.

## Figures and Tables

**Table 1:** Performance summary and comparison.

	[45]	[46]	[38]	This Work
Position sensing method	RSSI	TDoA	SRT	RSSI-assisted TDoA
Positioning error (m)	0.66 (average)	1.5 (the 90th percentile)	0.23 (RMS)	0.3 (the 90th percentile)
Offline phase	Complicated	None	None	Few
Computational complexity	o(n)	$o(n^{2})$	$o(n^{3})$	$o(n^{2})$
Real-time performance	+	-	–	-
